# Inflammation in Chronic Wounds

**DOI:** 10.3390/ijms17122085

**Published:** 2016-12-11

**Authors:** Ruilong Zhao, Helena Liang, Elizabeth Clarke, Christopher Jackson, Meilang Xue

**Affiliations:** 1 Sutton Arthritis Research Laboratory, Kolling Institute of Medical Research, University of Sydney, NSW 2065, Australia; rzha9073@uni.sydney.edu.au (R.Z.); helena.liang@sydney.edu.au (H.L.); chris.jackson@sydney.edu.au (C.J.); 2 Murray Maxwell Biomechanics Laboratory, Kolling Institute of Medical Research, University of Sydney, NSW 2065, Australia; elizabeth.clarke@sydney.edu.au

**Keywords:** chronic wound, inflammation, venous ulcer, arterial ulcer, pressure ulcer, diabetic ulcer, ageing, hypoxia, ischaemia-reperfusion, bacterial colonisation

## Abstract

Non-healing chronic wounds present a major biological, psychological, social, and financial burden on both individual patients and the broader health system. Pathologically extensive inflammation plays a major role in the disruption of the normal healing cascade. The causes of chronic wounds (venous, arterial, pressure, and diabetic ulcers) can be examined through a juxtaposition of normal healing and the rogue inflammatory response created by the common components within chronic wounds (ageing, hypoxia, ischaemia-reperfusion injury, and bacterial colonisation). Wound bed care through debridement, dressings, and antibiotics currently form the basic mode of treatment. Despite recent setbacks, pharmaceutical adjuncts form an interesting area of research.

## 1. Overview

The skin forms an important and effective barrier against the environment. It plays a vital role in protection against insults such as bacteria, xenobiotics and dehydration. When a cutaneous injury occurs, the body initiates a series of complex events to re-establish this protection. Wound healing can be roughly divided into four continuous and overlapping phases: (1) haemostasis; (2) an immediate inflammatory response defined by an infiltration of cytokine-releasing leukocytes with antimicrobial functions; (3) these cytokines kick off a proliferative phase where new epithelium, blood vessels, and extracellular matrix (ECM) are laid down; (4) over a period of weeks to months, the wound contracts as the ECM is remodelled [1]. These highly regulated cellular, humoral and molecular processes have been described as an orchestral performance—a potential flawless interplay can lead to perfect regeneration; however, human adult wounds undergo a repair process that leads to scarring, and, in some cases, non-healing chronic wounds [2].

Chronic wounds exhibit such a disrupted repair process where a sustained anatomical and functional result is not reached within an appropriate length of time—usually taken to be three months [3]. They fail to progress through the orderly phases of healing but are detained in a self-perpetuating inflammatory stage, and remain intractable despite adequate wound management. A myriad of factors can delay wound healing—chronic disease, vascular insufficiency, diabetes, malnutrition, ageing, and local factors such as pressure, infection, and oedema [4]. The subsequent tissue damage locks the wound in a prolonged and heightened inflammatory state characterised by abundant neutrophil infiltration, with its associated reactive oxygen species (ROS) and destructive enzymes perpetuating the cycle [5]. Eliminating the primary noxious factor, however, can effectively heal many chronic wounds [6].

Non-healing ulcers represent a major burden for patients and their families. They cause pain, infections, loss of function, and financial costs, and often lead to amputations or sepsis. Chronic wounds mostly arise as a problem secondary to high profile issues such as an ageing population, obesity and diabetes [7]. These health issues are rapidly increasing in most parts of the world, and, with that, the incidence of non-healing pressure, venous, and diabetic ulcers. Unfortunately, the significance of chronic wounds is overshadowed by their causes—its costs are poorly documented, and appropriate care and education is lacking [8]. However, it persists as a silent epidemic impacting the quality of life of over 40 million people worldwide [9].

## 2. Causes

The aetiology of chronic wounds is diverse, hindering the efforts of researchers to find a single therapeutic agent as a panacea of healing. The cellular and molecular mechanisms of wound healing are complex, and their impairments poorly understood. A diverse range of comorbidities present further hurdles in identifying therapeutic targets and the design of clinical trials. Adding to this is the paucity of suitable animal models of wound healing. For example, there is a relative absence of truly aged animals that can provide a precise model of chronic wounds [10]. Reproducing comorbid and causative conditions such as neuropathy, chronic debility or vascular insufficiency in animals is difficult—not to mention that most animals are loose-skinned, and their open wounds heal mostly by rapid wound contraction—a stark contrast to the lower limbs of humans, which are a common site for chronic wounds [11,12,13]. However animal models can provide insight into specific pathways, providing a focused approach on selected mechanisms underlying chronic wounds [14].

The vast majority of chronic wounds fall into three main categories: venous ulcers, pressure ulcers, and diabetic ulcers, with a smaller fourth group secondary to arterial ischaemia [10].

### 2.1. Venous Ulcers

Venous stasis ulcers account for more than half of all lower limb chronic wounds and will affect 1%–2% of the adult population, with a higher prevalence in women and the elderly [4]. They arise secondary to venous hypertension and congestion due to venous thrombosis or valvular incompetence. The backpressure increases blood vessel permeability, leading to leakage of macromolecules and red blood cells into the perivascular space, where they may act as chemoattractants for leukocyte infiltration [15,16]. Subsequent oedema and fibrosis impedes the diffusion of oxygen, growth factors and nutrients into the wound tissue [17]. This effective loss of circulation is restored with leg elevation, and the inflammatory processes associated with reperfusion exacerbate injury (discussed below) [10]. Venous ulcers tend to be larger and shallower, with typically irregular and ill-defined margins, most commonly occurring on the medial malleolus [4].

### 2.2. Arterial Ulcers

Arterial ulcers are less common than venous ones. They occur as a consequence of arterial insufficiency caused usually by atherosclerosis, or, more rarely, thromboemboli or radiation damage [18]. Narrowing of arterial lumen reduces perfusion, leading to ischaemia and hypoxia. Such an occlusion of arteries other than those that supply the heart or brain is defined as peripheral vascular disease, with major risk factors including smoking, diabetes, hypertension, hypercholesterolaemia, and a prevalence that increases with age [19]. Wound therapy involves management of risk factors and may require restoration of the peripheral flow with angioplasty or reconstructive surgery [20]. Arterial ulcers normally occur distally over bony prominences and present with a round, sharply demarcated border [20].

### 2.3. Pressure Ulcers

Pressure ulcers are common in patients with compromised mobility and sensory perception—paralysed or unconscious patients who can neither sense nor respond to the periodic need for repositioning. Prolonged unrelieved pressure or shear leads to ischaemia when tissue compression exceeds capillary pressure [4]. Necrosis results from tissue hypoxia and ischaemia-reperfusion injury [6]. Skin over bony prominences such as the sacrum, hips, and malleoli are especially vulnerable, often after as little as two hours of immobility [21].

### 2.4. Diabetic Ulcers

Diabetes mellitus is one of the leading causes of death, with over 382 million people living with the disease worldwide [22]. Diabetic foot ulcers are a common and serious complication of diabetes [23]. A pathogenic triad of neuropathy, ischaemia, and trauma is traditionally described [24]. Diabetes associated peripheral neuropathy creates a structurally weakened, insensate foot, increasing the risk of ulceration from repeated mechanical stress, compounded by disrupted perfusion [4]. Moreover, diabetes causes hyperglycaemia-related metabolic derangements that directly disrupt wound healing [23]. These include a systemic accumulation of advanced glycation end-products (AGEs) that induces oxidative stress, impairs skin and inflammatory cell function, and increases ECM stiffness, among others; a chronic low-grade and self-perpetuating inflammatory state; and micro- and macro-circulatory dysfunctions leading to poor oxygen perfusion [25,26,27,28] Patients with diabetic foot ulcers are at a higher risk of re-ulceration, amputation and death; such severe outcomes have warranted greater attention to diabetic wound healing and limb salvage over the last decade [22].

## 3. Normal Cutaneous Healing

An understanding of the underlying mechanisms involved in chronic wounds can be aided by a review of the well-regulated, predictable process of physiological wound healing. As the normal phases of wound healing have been extensively described [1,2], we focus here on inflammation.

### 3.1. Haemostasis

The first stage of healing is haemostasis and the formation of a provisional wound matrix. Following endothelial injury, components in the underlying ECM bind and activate circulating platelets, which then undergo adhesion and aggregation. Damaged tissue and aggregated platelets trigger the extrinsic and intrinsic coagulation pathways, respectively—working together to stabilise the fibrin-platelet clot. This forms a scaffold for the migration and proliferation of other cells involved in wound healing, as well as a reservoir for cytokines and growth factors.

### 3.2. Inflammation

Inflammation is an essential, nonspecific, innate immune response involving the breakdown of tissue and clean-up of cellular, extra-cellular and pathogen debris. Normally, this resolves after one to two weeks when infiltrated leukocytes return to their pre-inflammation numbers and phenotypes [29]. However, in the presence of an external noxious stimulus causing tissue damage, inflammation can become prolonged and heightened.

Both platelets and leukocytes release inflammatory cytokines that provide a chemotactic gradient for additional leukocytes to enhance the inflammatory process. These include interleukin (IL)-1α, IL-1β, IL-6, IL-8, tumour necrosis factor (TNF)-α, platelet-derived growth factor (PDGF) and transforming growth factor (TGF)-β. Notably, PDGF plays an important role in initiating the chemotaxis of neutrophils, monocytes, smooth muscle cells and fibroblasts, whereas TGF-β stimulates cytokine secretion from macrophages and enhances fibroblast and smooth muscle cell chemotaxis [5]. The net result is a vigorous response of matrix-producing cells ensuring a rapid deposition of connective tissue for the highly cellular phases of inflammation and proliferation. This recruitment is helped by a primarily histamine-mediated vasodilation, a process that takes over vasoconstriction around 10 min after wounding [30]. Histamine from mast cells creates pores in blood vessels, facilitating protein leakage and extravasation of leukocytes into the wound site.

Initial leukocyte response is dominated by neutrophils for the first two to five days, with macrophages taking over from approximately day three. Neutrophils have three main functions. Firstly, they generate free radicals via the myeloperoxidase pathway to kill bacteria [6]. They also debride the wound through secretion of proteolytic enzymes that break down nonviable tissue. These include proteases with broad substrate specificity such as serine proteases, or with narrow specificity such as matrix metalloproteinase (MMP)-2 and -9 that degrade collagen and collagen fragments [31]. Lastly, neutrophils phagocytose the dead bacteria and matrix debris. They usually undergo apoptosis once their tasks are completed and are mopped up by macrophages.

As monocytes immigrate into the wound and mature into macrophages, they become the most important regulatory cell in the inflammatory reaction [1]. The highly phagocytic macrophages remove any non-functional host cells, bacterial-filled neutrophils, damaged matrix, foreign debris, and remaining bacteria [5]. Activated macrophages secrete further growth factors, chemokines and cytokines such as TGF-α, TGF-β, basic fibroblast growth factor (bFGF), PDGF, and vascular endothelial growth factor (VEGF) to amplify and eventually resolve inflammation—recruiting endothelial cells and fibroblasts to initiate the next phase of healing. While inflammation is vital in clearing infection and debris, it can lead to tissue damage if prolonged. This may result in increased scarring or chronic wounds.

### 3.3. Proliferation

In the proliferative phase, cellular activity predominates in response to increased levels of cytokines such as epidermal growth factor (EGF), VEGF and TGF-β [32]. Re-epithelialisation commences as keratinocytes and epithelial stem cells proliferate and migrate over a fibrin/fibronectin-rich provisional ECM [2,33]. As inflammation wanes, dermal reconstitution gets underway, and endothelial cells and fibroblasts begin to accumulate in the wound site to synthesise granulation tissue [1]. Angiogenesis and fibroplasia occur simultaneously, providing oxygen, nutrients, and a very hydrated matrix to sustain the high levels of cellular activity. Fibroblasts play an important role in producing the ECM substances of this matrix (collagen, fibronectin, glycosaminoglycans, proteoglycans and hyaluronic acid), which interact with cells to mediate migration, growth, and differentiation [34,35].

### 3.4. Remodelling

Contraction and remodelling is the last phase of wound healing. In response to mechanical tension and cytokines such as TGF-β, fibroblasts increase expression of *α*-smooth muscle actin, transforming into myofibroblasts, which contract the wound via their integrin receptor contacts to ECM components such as fibronectin and collagen [32,36]. Remodelling occurs as fibroblasts upregulate the expression of the stronger type I collagen, and MMPs breakdown disorganised old collagen, primarily of type III. A tightly controlled balance between synthesis and lysis of collagen results in the development of a normal scar where collagen fibres are rearranged in small parallel bundles along tension lines, once again predominantly of the type I phenotype [2,37]. The end of healing is heralded by apoptosis of vascular cells and myofibroblasts as their work is completed, converting the cell rich granulation tissue into a collagen filled, hypocellular scar [38].

### 3.5. Necessity of Inflammation

It has been hypothesised that evolutionary pressures have driven the need for an intense inflammatory healing response for medium-sized dirty wounds—irregular tears, blunt trauma and bites—such injuries sustained by primitive people and animals are almost guaranteed to be contaminated, thus keeping the wound open longer until inflammatory cells have cleared the foreign microorganisms was favourable [39,40]. However, in modern, sterile surgical wounds, such a response is unwarranted and unwanted—not only does it delay healing, scars create unaesthetically-pleasing, weaker skin [39].

While the skin of mammals mostly heals via tissue repair (tissue adaptation after injury with scar tissue), regeneration refers to replacement with an exact copy of the tissue that was damaged, such that both morphology and function are completely restored [41,42]. Complete regeneration is limited to invertebrates and lower vertebrates such as salamanders [43]. However, scarless cutaneous wound healing has also been observed in the foetuses of mammals such as rats, mice, pigs, monkeys and humans [44,45]. Evidence suggests that regeneration is, in fact, due to differences intrinsic to foetal tissue such as cytokines, cellular and ECM mediators, and, in particular, a reduced inflammatory response [46]. Perhaps from a thorough understanding of the precise differences in foetal wound inflammation, we can learn to artificially alter the ratio of various factors involved in adult healing, in order to induce wounds that also heal perfectly free of scarring and to eliminate wound chronicity.

## 4. Pathophysiology

Chronic wounds do not follow such a well-defined cascade. They are often locked in an inflammatory state that precludes proliferation. Among others, local tissue hypoxia, repetitive trauma and heavy bacterial burden, combined with impaired cellular and systemic host responses to stress perpetuate a deleterious cycle that prevents progression into the proliferative phase of healing [47]. The high level of mitogenic activity seen in acute wounds is absent in chronic wounds [47]. The often multifactorial stimuli create and amplify a hostile microenvironment where the delicate balance between pro-inflammatory cytokines, chemokines, proteases, and their inhibitors that exists in acute wounds is disturbed [48]. As such, the injury fails to heal within a physiologically appropriate length of time. This delay in wound healing also exacerbates scarring due to prolonged inflammation, and is predisposed to neoplastic progression [49,50].

Excessive neutrophil infiltration appears to be a critical culprit in this cycle of chronic inflammation, and acts as a biological marker of chronic wounds [5]. Abundance of neutrophils leads to over-production of ROS, causing direct damage to ECM, cell membrane and subsequently, premature cell senescence [15]. In addition, neutrophils release serine proteases such as elastase and MMPs such as neutrophil collagenase (MMP-8); elastase degrades important growth factors such as PDGF and TGF-β, while collagenase degrades and inactivates components of the ECM [5,15]. Hence, although growth factor production is often increased in chronic wounds, their bio-availability is decreased. Both neutrophils and activated macrophages also produce pro-inflammatory cytokines such as IL-1β and TNF-α that not only increase MMP production but also reduce tissue inhibitors of MMPs (TIMPs); this imbalance augments degradation of the ECM, impairs cell migration, and reduces fibroblast proliferation and collagen synthesis [51]. The ECM breakdown products further promote inflammation, creating a self-sustaining process. Such an inappropriate inflammatory response combines with the impaired cellular and systemic host response to stress, perpetuating the deleterious cycle that must be broken through debridement and cleaning of the wound for healing to occur.

**Figure 1:**
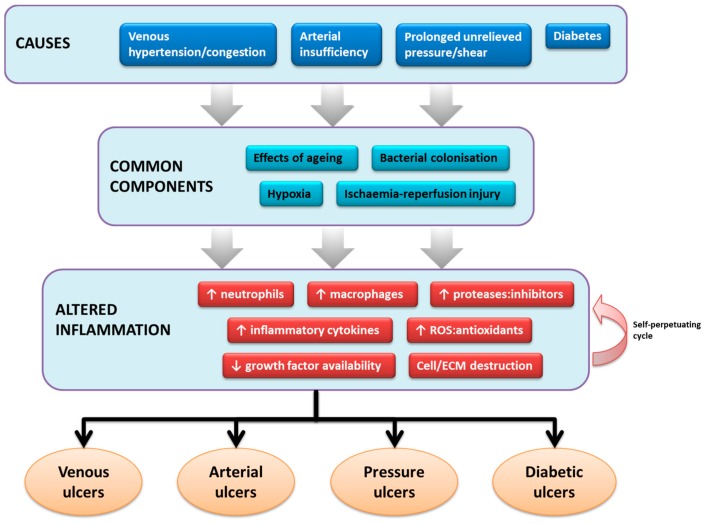
The role of inflammation in the pathophysiology of common chronic wounds. ROS = reactive oxygen species, ECM = extracellular matrix.

Despite different underlying causes, many chronic wounds behave and progress similarly, as summarised in Figure 1. Mustoe proposed that this uniformity is largely explained by the coexistence of several consistent components: cellular and systemic changes of ageing, repeated ischaemia-reperfusion injury in the context of local hypoxia, and bacterial colonisation [10]. They derange various healing processes—inflammation among them—instigating chronicity.

### 4.1. Ageing

With an ageing population, more emphasis must be placed on elucidating the age-related impairments in wound healing. Delayed wound healing in the elderly presents both a major clinical and economic problem, especially as most chronic wounds occur in this population [52]. Although the healing response in aged subjects is slower, animal and human studies have shown that the final result is comparable in quality to young subjects [53,54]. Specific age-related changes in inflammation include alterations in cell-adhesion, migration, and functional responses [55]. Ageing is associated with an early influx of neutrophils, which have significantly reduced respiratory burst activity, diminished ability to phagocytose bacteria, reduced CD16 expression, and impaired chemotaxis [55]. An abundance of neutrophils produces excess proteases, in particular elastase, which also degrades important structural and functional proteins such as proteoglycans, collagen, and fibronectin—leading to characteristically reduced local levels of fibronectin in age-related wounds [56]. Evidence suggests that while macrophage adhesion to substrates increases with age, there is delayed infiltration with an altered ratio of mature to immature populations, which may explain the overall reduction in phagocytic capacity [55,57,58]. Furthermore, activation and proliferation of lymphocytes is compromised in older individuals [59]. These changes all occur on a background of underlying inflammation as part of the ageing process, with upregulation of inflammatory cytokines including nuclear factor (NF)-κB, IL-1β, IL-6 and TNF-α [55].

### 4.2. Hypoxia

Many chronic wounds occur on a background of local tissue hypoxia due to vasculopathies such as atherosclerosis and venous hypertension, or periwound fibrosis which reduces perfusion. Local tissue hypoxia is well known to profoundly disrupt wound healing. Through various molecular mechanisms, hypoxia leads to cell membrane disruption, promoting inflammatory cascades [60]. The subsequent extravasation of neutrophils and macrophages is helped by a pronounced expression of endothelial adhesion molecules in hypoxic tissue; recruited neutrophils and macrophages then synthesise pro-inflammatory cytokines such as IL-1α, IL-1β, IL-6 and TNF-α in an autocrine manner [61]. As previously mentioned, this upsets the balance between proteases and their inhibitors, perpetuating inflammation. A balance between ROS and antioxidants is similarly disrupted. Various antioxidants such as nitric oxide (NO) are produced in an oxygen-dependent manner, and so decrease in hypoxic states [62]. NO is further involved in switching off NF-κB, an important transcriptional activator of inflammation [63]. Unchecked production of ROS not only causes oxidative damage, but also stimulates signal transduction pathways leading to enhanced expression of serine proteases, MMPs, and inflammatory cytokines [52]. The result of hypoxia is a potentiation of the inflammatory state, hindering healing. Hypoxia also affects other metabolic activities besides inflammation—re-epithelialisation is impaired, as is fibroblast proliferation and its synthesis of collagen [52].

### 4.3. Ischaemia-Reperfusion Injury

Ischaemia-reperfusion injury has been implicated in the pathophysiology of myocardial infarction, haemorrhagic shock, stroke, and organ transplantation [64]. It is also postulated to be the precipitating event for all three major aforementioned chronic wound types [10]. Vasculopathic patients with suboptimal circulation endure cyclic intervals of ischaemia in their lower limbs during leg use, followed by reperfusion during leg elevation. Ischaemia with subsequent tissue hypoxia induces a pro-inflammatory state, as stated above. During reperfusion, an over-abundance of leukocytes including neutrophils migrate into the wound tissue; there, they produce inflammatory cytokines and ROS, compounded by ROS from partial tissue reoxygenation [52]. NO is also downregulated, further accentuating inflammation [10]. These ischaemia-reperfusion cycles are repetitive, their deleterious effects are exacerbated, and are eventually sufficient to cause tissue necrosis and ulceration [10].

### 4.4. Bacterial Colonisation

The third pathogenic factor in chronic wounds is obligate bacterial colonisation. Common wound pathogens such as *Staphylococcus aureus*, *Pseudomonas aeruginosa* and β-haemolytic streptococci specifically cause delayed healing [65]. Besides direct damage to the host, bacteria attract leukocytes with resultant amplification of inflammatory cytokines, proteases, and ROS—thus both initiating and maintaining inflammatory cascades [52]. Host and bacteria derived proteases and ROS degrade ECM and growth factors, disrupting cell migration and inhibiting wound closure [15]. Bacteria that colonise chronic wounds often form polymicrobial biofilms, in contrast to a minimal incidence within acute wounds [66]. Microbial cells are embedded in a secreted polymer matrix, which provides an optimal environment for bacteria to evade host immune response and antibiotic action [67]. Although the association of biofilms with chronic wounds is confirmed, causality remains an evolving topic. Microbial synergy within a biofilm provides a competitive advantage to cohabiting organisms, but little is known about how this synergy may increase the net pathogenicity in chronic wounds [65]. Indeed, it has even been suggested that the entrenched biofilm precisely commandeers the host inflammatory response, protracting it to obtain a sustainable nutrient source in the form of inflammatory exudate [68]. Hypoxia within the wound bed also contributes to bacterial colonisation. Numerous studies show an inverse correlation between infection and wound oxygenation, likely due to the oxygen-dependent activity of antimicrobial enzymes such as myeloperoxidase within neutrophils [10,52]. Hence, periods of ischaemia in chronic wounds hinder host bactericidal mechanisms. Several landmark studies have demonstrated that a bioburden exceeding 10^5^ bacteria per gram of wound tissue has a deleterious effect on healing in both various acute and chronic wounds, as well as skin grafts [47].

## 5. Current Treatments

Chronic wounds are complex and inflammatory in nature. Pro-inflammatory stimuli such as necrotic tissue, a heavy bacterial burden, and tissue breakdown lead to copious amounts of exudate, which disrupts normal healing and interferes with advanced therapeutic products [69]. Here in the local wound milieu, cellular and biochemical changes serve as primary targets of chronic wound management, regardless of the underlying cause [14]. In 2002, the International Wound Bed Preparation Advisory Board developed a systematic and structured approach to such management [69]. Barriers to healing were identified according to the TIME acronym for adequate wound bed preparation—tissue, non-viable or deficient, infection or inflammation, moisture imbalance, and non-advancing or undermined epidermal margin or edge. Once recognised, the offending conditions could be removed through debridement of the necrotic burden, management of exudate with dressings, and resolution of bacterial imbalance with topical and systemic antibiotics. These and other basic wound management tenets such as tissue oxygenation and adequate nutrition remain crucial components to aiding a proper healing response [70]. Obviously, a comprehensive assessment to address the underlying aetiology is also necessary [47].

The ultimate aim is termination of inflammation by removing perpetuating causes, and provision of an optimal environment for healthy granulation formation, with a view to transform the chronic wound inflammatory characteristics into that of an acute wound. However, the complexity and heterogeneity of wounds have hindered novel pharmacological attempts as adjuncts to wound bed preparations to modify the wound characteristics. Hence, dressings and devices have remained the mainstay of wound care, despite many lacking clinical evidence [71]. Early interest in the therapeutic roles of exogenous growth factors and cytokines has diminished after lacklustre performances in clinical trials [70]. Nevertheless, there is great potential in this area as scientific knowledge advances and techniques become more sophisticated.

Kerotinocytes, endothelial cells, fibroblasts and resident immune cells synthesise, and are stimulated by, cytokines and growth factors. The complex, coordinated efforts of skins cells in the cutaneous healing cascade is regulated by an equally intricate signalling network [72]. As the cells undergo changes in a wound, so too does their cytokine/growth factor profile [71]. Thus, modifying their levels in chronic wounds to match those in an acute wound can extinguish inflammation and kick-start proliferation. Many experimental and clinical studies have revealed varied, but mostly beneficial effects of exogenous cytokines and growth factors [32]. Due to their multifaceted roles, involved biomolecules often affect multiple pathways and phases of healing, more than simply reducing inflammation. In 1997, PDGF, or becaplermin, became the first and only US Food and Drug Administration (FDA) approved growth factor to be used in wound healing under the trade name Regranex, specifically for diabetic foot ulcers. PGDF is chemotactic for neutrophils and macrophages, and hence plays an important role in the inflammatory response. Clinically, however, becaplermin has demonstrated only modest improvements in healing diabetic and pressure ulcers, but it and other growth factors have been used off-label in various acute and chronic wounds to some degree of success [70]. Recently, an angiotensin receptor agonist called aclerastide—also for the treatment of diabetic foot ulcers—advanced to phase III clinical trials, where, unfortunately, the study was terminated after conducting futility determinations [73]. Other growth factors, such as bFGF and VEGF, have similarly shown mixed clinical results despite promising in vitro and animal studies [74,75,76,77,78,79,80].

## 6. Future Prospects

Despite the clear, unmet clinical need for new targeted therapies, the intricacy of chronic wounds and their multifactorial causes has precipitated a current reality relatively barren in wound healing drugs than predicted [10]. A lack of funding, limitations of preclinical animal models, and the inherent difficult nature of complete closure of chronic wounds as the only primary outcome in clinical trials all contribute to the slow research advancement [14]. It may be unlikely that a single therapeutic target will be highly efficacious; the challenge probably lies in combination therapy and appropriate delivery vehicles to circumvent issues of short half-lives within the hostile inflammatory microenvironment rich in proteases [81].

Another class of biochemical substances is characterised by their pleiotropic functions. Unlike cytokines and growth factors that activate or inactivate various processes, such biomolecules have diverse effects that modulate respective pathways. Rather than adjusting the levels of merely one cell type, cytokine or growth factor in the vastly complex system of interacting cellular, humoral and molecular processes, it may be this modulation that provides the answer to improving wound healing [82]. A notable example is activated protein C (APC), which was first discovered for its more well-known activities in anticoagulation but has since demonstrated potent pleiotropic anti-inflammatory and cytoprotective properties [83]. A number of preclinical and pilot clinical trials have demonstrated very promising results to support APC’s use in chronic wounds [84,85,86,87,88,89,90,91].

However, based on questions raised about APC’s potential bleeding side effects, there have been encouraging developments in engineered APC that minimise its side effects, and peptides that mimic their positive actions [92,93,94].

## 7. Conclusions

The consequences of chronic wounds create an unfortunate reality of handicap and frustration for their sufferers. Patients and doctors alike will no doubt benefit from a deeper understanding of the pathogenesis and pathophysiology of various non-healing ulcers. New insights have been derived from a comparison of the similarities between the chronic wounds of diverse aetiologies, contrasted with their differences from acute wounds. Unbridled inflammation in particular, has been implicated in playing a pivotal role. As such, resolving the inflammatory milieu has been a target of both traditional wound care and experimental drug-based therapies. Despite formidable challenges in this exciting area of research, new developments, such as the pleiotropic APC, may lead to a future where we can better modulate the cytokine and growth factor profile of chronic wounds, minimising a massive healthcare burden.

## Abbreviations

AGE	advanced glycation end-products
APC	activated protein C
ECM	extracellular matrix
EGF	epidermal growth factor
FDA	Food and Drug Administration
FGF	fibroblast growth factor
IL	interleukin
MMP	matrix metalloproteinase
NF	nuclear factor
NO	nitric oxide
PDGF	platelet-derived growth factor
ROS	reactive oxygen species
TGF	transforming growth factor
TIMP	tissue inhibitor of matrix metalloproteinase
TNF	tumour necrosis factor
VEGF	vascular endothelial growth factor
